# Effects of Implementing Personalized Health Education in Ambulatory Care on Cardiovascular Risk Factors, Compliance and Satisfaction with Treatment

**DOI:** 10.3390/jpm12101583

**Published:** 2022-09-26

**Authors:** Agata Bielecka-Dabrowa, Joanna Lewek, Agata Sakowicz, Aleksandra Paduszyńska, Marek Dąbrowa, Daria Orszulak-Michalak, Maciej Banach, CARDIOPLUS Investigators

**Affiliations:** 1 Department of Preventive Cardiology and Lipidology, Medical University of Lodz, 93-338 Lodz, Poland; 2 Department of Cardiology and Congenital Diseases of Adults, Polish Mother’s Memorial Hospital Research Institute (PMMHRI), 93-338 Lodz, Poland; 3 Department of Medical Biotechnology, Medical University of Lodz, 90-752 Lodz, Poland; 4 Department of Biopharmacy, Medical University of Lodz, 90-151 Lodz, Poland; 5 Cardiovascular Research Centre, 65-417 Zielona Góra, Poland

**Keywords:** prevention, personalized health education, cardiovascular risk factors

## Abstract

Aim and Methods: Data from the CARDIOPLUS study (a prospective, multicenter, non-interventional study, which was conducted among patients and physicians from ambulatory patient care in Poland) were used to assess whether primary care behavioral counseling interventions to improve diet, increase physical activity, stop smoking and reduce alcohol consumption improve outcomes associated with cardiovascular (CVD) risk factors, metabolic parameters, compliance and satisfaction with treatment in adults. The study was carried out throughout Poland in the period from July to December 2019. Results: The study included 8667 patients—49% women and 51% men aged (63 ± 11 years)—and 862 physician-researchers. At the 3-month follow-up, there was a significant reduction in body weight (*p* = 0.008); reduction of peripheral arterial pressure, both systolic (*p* < 0.001) and diastolic (*p* < 0.001); reduction in total cholesterol levels (*p* < 0.001), triglycerides (*p* < 0.001), and LDL cholesterol (*p* < 0.001). The percentage of respondents who fully complied with the doctor’s recommendations increased significantly. The respondents assessed their own satisfaction with the implemented treatment as higher (by about 20%). Conclusions: As a result of pro-health education in the field of lifestyle modifications, a significant reduction of risk factors for cardiovascular diseases, as well as improved compliance and satisfaction with pharmacological treatment, was observed. Thus, appropriate personalized advice on lifestyle habits should be given to each examinee in a positive, systematic way following the periodic health check-ups in order to reduce the person’s risk and improve the effectiveness of the treatment.

## 1. Introduction

Cardiovascular diseases (CVD) are responsible for more than 4 million deaths each year across Europe [1]. The number of deaths from CVD is higher in women (2.2 million) than in men (1.8 million), with CVD accounting for 49% of all deaths in women and 40% of all deaths in men [1]. Those data emphasize the need for prophylaxis both in the general population and individual patients. Studies show that the implementation of preventive actions can prevent more than 80% of CVD cases. Education and prevention should be tailored to the needs and capabilities of a particular patient, and the physician plays a key role in this process. As cardiovascular risk increases in patients, lifestyle modification and counseling should be intensified, and pharmacotherapy should be initiated, especially in very high-risk patients [2].

Studies show that programs of lifestyle change that include nutritional education and physical exercise might be efficient in achieving the proposed goals for the treatment of metabolic syndrome (MetS) [3,4]. In the study of Saboya P et al., the authors tried to identify whether there is some influence of performed behavioral counseling on the improvement process of the metabolic condition in 72 patients with MetS aged 30–59 years. Lifestyle modifying interventions resulted in a significant reduction in body mass index, waist circumference, systolic blood pressure at 3 months, and the improvement of QOL [5].

The most recent guidelines of the American College of Cardiology (ACC)/American Heart Association (AHA) [6] and the European Society of Cardiology [2] recommend the following non-pharmacological interventions for the control of diseases such as hypertension, diabetes, and lipid disturbances: restricted intake of alcohol, weight loss, intensification of physical activity with a structured exercise program, use of the Dietary Approaches to Stop Hypertension (DASH) diet, high intake of fruit and vegetables [6,7].

Positive health-promoting behavior, including lifestyle factors (healthy diet, smoking cessation, regular exercise, weight control), should be strongly advised [2].

Our focus on counseling interventions that take place in or were considered feasible for primary care among adults without risk factors for CVD or known CVD is relatively narrow. Thus, the aim of this study was to test whether a simple program of education and motivation in primary care about lifestyle change is effective in the reduction of cardiovascular risk factors, including metabolic parameters, as well as compliance and satisfaction with treatment in the population of Poland.

## 2. Methods

The observational study involved 8667 ambulatory care patients—49% women and 51% men aged (63 ± 11 years)—and 862 doctor-researchers.

Data from the CARDIOPLUS study were used to assess whether primary care behavioral counseling interventions to improve diet, increase physical activity, stop smoking, and reduce alcohol consumption improve outcomes associated with CVD risk factors, metabolic parameters, compliance, and satisfaction with treatment in adults.

CARDIOPLUS was a prospective, multicenter, non-interventional study that was conducted among patients and physicians from ambulatory patient care in Poland, assessing tolerability and satisfaction with treatment with acetylsalicylic acid (ASA) 100 mg). The study was carried out throughout Poland in the period from July to December 2019. The aspirin treatment was not initiated as a part of the study, and the included patients were on acetylsalicylic acid regardless of the study participation.

Inclusion criteria were defined as:

- Age ≥ 18 years,
- Psychophysical state of health, which promises compliance with medical recommendations,
- Treatment for up to 4 weeks with acetylsalicylic acid at a dose of 100 mg.

Exclusion criteria were as follows:

- Chronic mental illnesses,
- Alcohol and/or drug addiction,
- Contraindications to the use of drugs containing acetylsalicylic acid.

The study began on May 2019, when the materials were distributed to the physicians. In May 2019, the recruitment process started; the second visit took place between September and October 2019. Completion of patients’ data was on October 2019.

Each of the doctor-researchers participating in the study conducted it with at least 10 patients who met all inclusion criteria for the study.

In order for the study to be considered completed by a given doctor-investigator, it was required to conduct the study in its entirety, i.e., with the entire group of patients enrolled in it and within the prescribed period.

The tool used to carry out the study was a two-visit questionnaire. Study data were collected through a standardized list of questions (“questionnaires”) to be completed by a physician based on patient information, personal observations, physical examination, and lipid profile results. The study was aimed at both doctors and patients from all over the country. The study consisted of two parts: an interview questionnaire completed during the patient’s first visit to the doctor’s office and the second questionnaire conducted during the next visit (approximately 12 weeks (±2weeks) after the first visit, depending on the physician’s decision).

The interview questionnaire in the “first visit” consisted of questions specifying the patient’s health condition at the time of inclusion in the study and questions regarding the therapy—the fact of tolerance, satisfaction with therapy, compliance, and lipid profile results. In addition, during the first visit, the researcher provided non-pharmacological recommendations for the patient. The interview questionnaire in the “second visit” part consisted of questions specifying the patient’s health condition during the study and questions about the therapy—satisfaction with therapy, therapy tolerance, compliance, degree of patient implementation of non-pharmacological recommendations on CVD risk factors, and control lipid profile results. At the second visit, we assessed compliance with the non-pharmacological recommendations provided to patients by the researcher during the first visit.

Ambulatory care physicians conducted individual personalized consultations during the first and second visits with patients focusing on the prevention of cardiovascular diseases according to the guidelines, including increasing physical activity (to at least the recommended level of 150 min/week), diet modification, and weight reduction, as well as limiting alcohol consumption and smoking cessation [2,8,9,10]. The patients were also given educational leaflets covering health issues to read at home about hypolipemic diet with menu proposition, proper physical activity, and methods of reducing cardiovascular risk factors.

The diet program was based on the healthy diet model, which included the consumption of 30–45 g of fiber, 200 g of fruit, and 200 g of vegetables per day. Patients were advised to consume fruit and vegetables for 2–3 servings per day and fish at least twice a week [8,11].

The general practitioners (GP) advised patients to avoid alcohol, and those who drink regularly were advised to consume no more than 20 g per day for men and 10 g per day for women.

Patients were also asked to rate their satisfaction with the treatment on a 10-point scale, where 1 meant a very low grade and 10 very high. A score of 8–10 meant high to very high satisfaction with the treatment. As part of the questionnaire, GPs determined the level of achievement of the goals set for the currently used pharmacotherapy by selecting one of the three responses: full implementation, partial implementation, or no implementation.

All patients gave informed consent prior to the procedure. The study protocol is in compliance with the Declaration of Helsinki and was approved by Bioethics Committee of the Polish Mother’s Memorial Hospital Research Institute in Lodz (opinion number 56/2019 (RNN///KE).

## 3. Statistical Analysis

The statistical analyses were performed by the use of Statistica software (v13.1 Statsoft, Poland). The distribution of continuous data was analyzed by the two-tailed Student *t*-test between two different groups or *t*-test for dependence groups (the first and the second visit). The categorical data were analyzed by the chi-square test. All results were considered significant for *p* < 0.05.

## 4. Results

**Figure 1:**
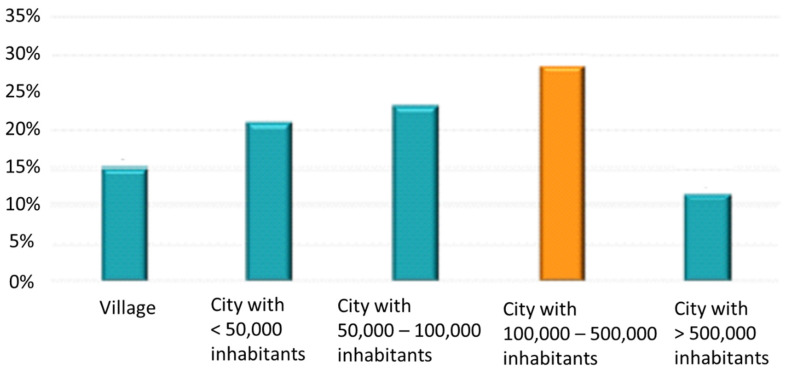
Population distribution according to the place of living.

**Figure 2:**
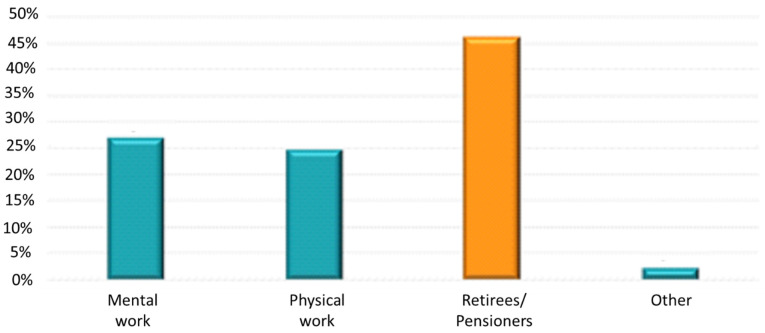
Type of professional activity of studied population.

**Figure 3:**
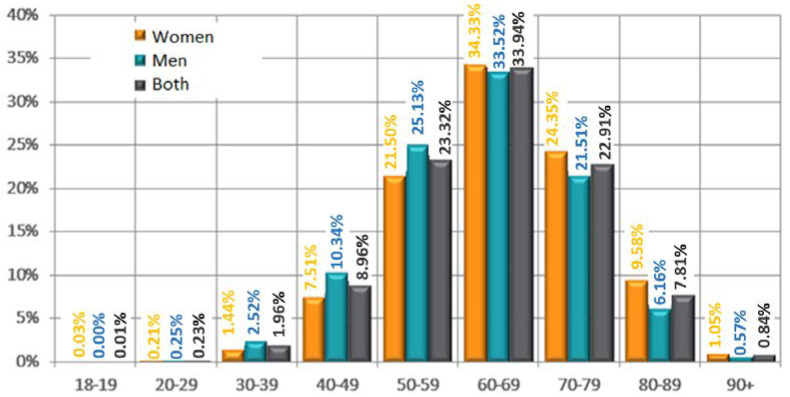
Age groups according to sex.

City residents constituted 84.7% of the total study population—Figure 1. Among them, the highest percentage lived in cities with >100,000–500,000 inhabitants (28.6%). Almost half of the patients were retirees/pensioners (46.2%)—Figure 2. The mean body mass index (BMI) was 29 ± 4.7 kg/m^2^. The detailed characteristics of age and BMI in the analyzed population are presented in Table 1. The age groups and BMI of the respondents according to gender are presented in Figure 3.

**Figure 4:**
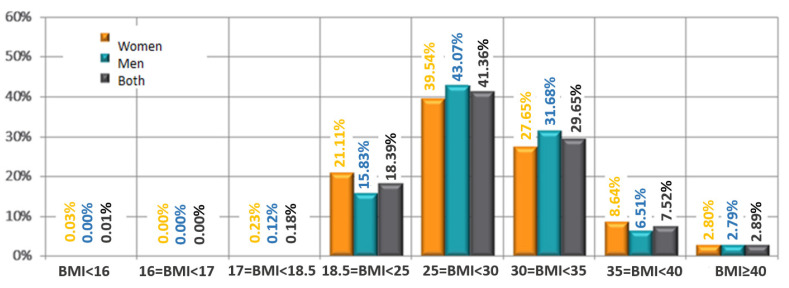
Distribution of BMI in studied population according to sex.

**Figure 5:**
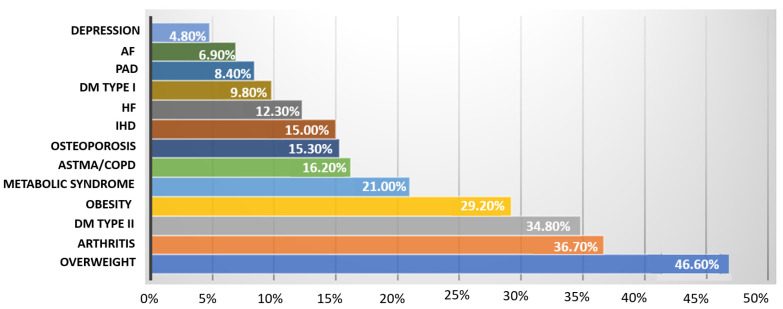
Comorbidities (IHD—ischemic heart disease; HF—heart failure; AF—atrial fibrillation; PAD—peripheral arterial disease; DM—diabetes mellitus; COPD—chronic obstructive pulmonary disease).

**Figure 6:**
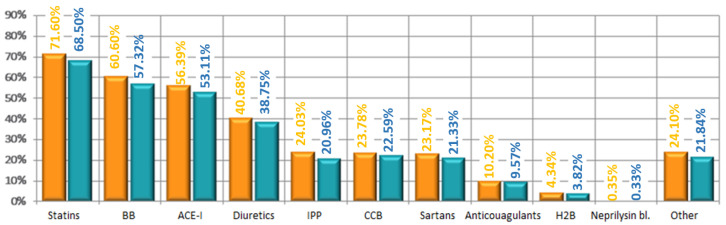
Pharmacotherapy in studied population (visit 1–visit 2) (BB—beta-blockers; ACE-I—angiotensinconverting enzyme inhibitor; IPP—inhibitor of proton pomp; CCB—calcium channel blockers; H2B—H2 receptor blockers; bl.—blockers).

**Figure 7:**
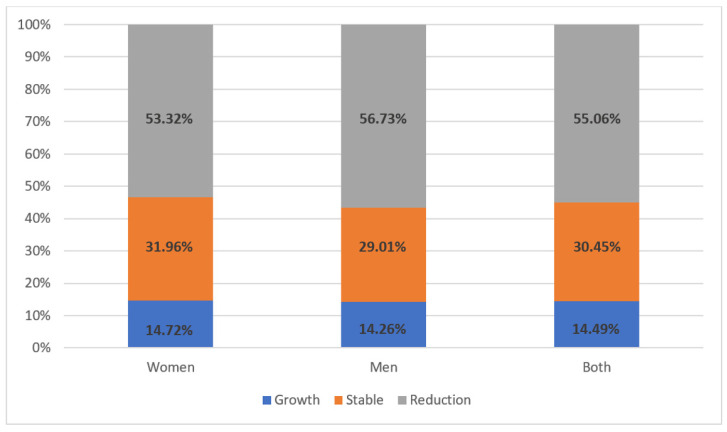
Change in body weight in studied population according to gender (visit 1–visit 2).

Arterial hypertension was diagnosed in 65.70% of the study participants over the years 1955/2019, including half of the cases in the past 5 years. Mean systolic blood pressure in the whole study population was 138 ± 14 mmHg, and diastolic blood pressure was 84 ± 9 mmHg. Regarding other comorbidities and disturbances, 8.5% had a stroke, 14.6% had a heart attack, and 12.3% had heart failure in the interview. Being overweight was the most common disease in the studied population (46.6%)—Figure 4. About 1/3 of patients had osteoarthritis, diabetes, or obesity (36.7%; 34.8%; 29%, respectively)—Figure 5. Twenty-seven percent of patients reported current smoking on the first visit, and 25% used to smoke in the past. The most common drugs used in the study were statins, beta-blockers, ACE inhibitors, and diuretics, without significant differences between the first visit and the follow-up visit (Figure 6). At the follow-up visit, there was a significant reduction in body weight (55.06% versus 45%; *p* = 0.008) (Figure 7).

**Figure 8:**
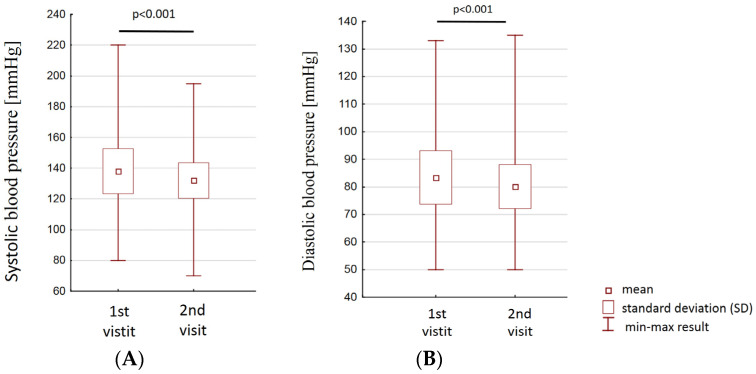
Comparison of blood pressure (**A**)—systolic; (**B**)—diastolic between the first and second vistits.

There was also observed a reduction in peripheral arterial pressure, both systolic (138 ± 14 versus 132 ± 11 mmHg; *p* < 0.001) and diastolic (80 ± 8 mmHg vs. 84 ± 10; *p* < 0.001)—Figure 8.

**Figure 9:**
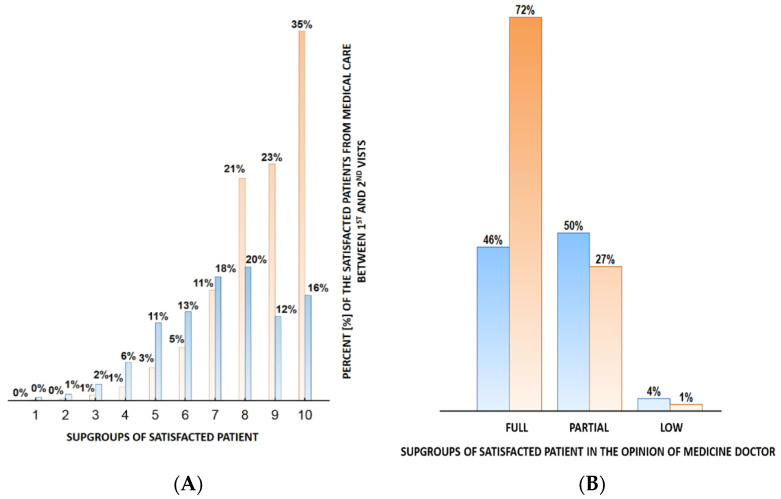
The comparison of the satisfaction level of the patients from the medical care between first and second visit. Blue graph—first visit; orange graph—second visit. Graph (**A**) represents the percent of the satisfied patients calculated for each subpopulation separately. The subpopulations of the patients were created according to the subjective opinion of the patients: 1—the lowest satisfaction up to 10—the highest satisfaction. Graph (**B**) represents the percent of satisficed patients according to the opinion of the medicine doctor. The percent of the satisfied patients were calculated separately for each of the following subgroups: full, partial and low.

**Figure 10:**
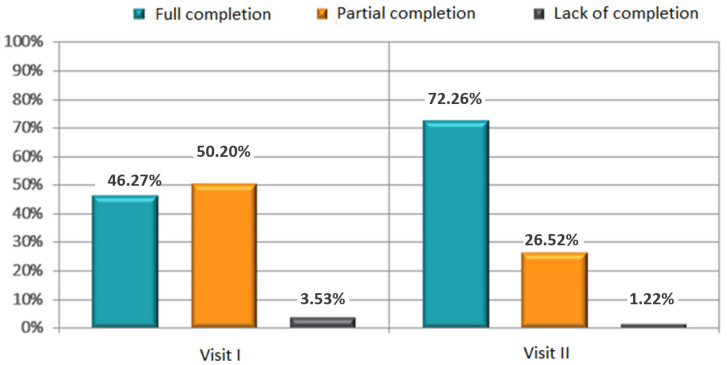
Subjective assessment of satisfaction with the treatment—completion of pharmacotherapy targets in the opinion of the doctor (*p* < 0.001).

At the follow-up visit, the concentrations of total cholesterol (196 ± 38 mg/dL versus 209 ± 44; *p* < 0.001), low-density lipoprotein cholesterol (LDL-C) (111 ± 33 mg/dL versus 121 ± 39, *p* < 0.001) and triglycerides (141 ± 49 mg/dL versus 155 ± 69; *p* < 0.001) had also decreased (Table 2). There was no significant difference in high-density lipoprotein cholesterol (HDL-C) after follow-up. The parameters of lipid profile at baseline and 3 months after lifestyle counseling are presented in Table 3. The percentage of respondents who reported a high to a very high level of satisfaction with the treatment provided was 62.8% (score of 8–10) (Figure 9, Figure 10). The percentage of respondents who fully complied with the doctor’s recommendations increased significantly. The respondents assessed their own satisfaction with the implemented treatment as higher (by about 20%).

## 5. Factors Influencing Medical Adherence to Lifestyle Modification

In general, the inhabitants of small towns (50–100,000 inhabitants) follow the recommendations the best (over 85% vs. 80% in villages and larger towns; *p* = 0.01), and the worst was in patients aged 70–90 years (75% vs. 85% in younger age groups; *p* = 0.00001) as well as old age and retired (79% vs. 85% compared to the employed; *p* = 0.0002). The gender and type of work performed did not have statistical significance.

### 5.1. Physical Activity

Regarding the recommended physical activity, the best adherence to the recommendations of physical activity was observed in patients with normal body weight and overweight but not obese (83% vs. 79%, *p* = 0.0006). Higher values of TCh and LDL cholesterol motivated patients to increase physical activity (patients with TCh > 190—almost 83% vs. 80% in patients with TCh < 190, *p* = 0.019; and patients with LDL > 100—over 83% vs. 80% in patients with LDL < 100, *p* = 0.006).

### 5.2. Body Weight-Diet Reduction

Patients over 50 years of age, and especially those over 70, presented poor compliance with dietary recommendations (67.7% of patients aged over70 years vs. 79% of patients under 49 years of age, *p* = 0.007). Gender, smoking, arterial hypertension, and lipid profile values had no effect on dietary adherence. The best adherence to the dietary recommendations was observed among inhabitants of cities with a size of 50–100,000, the worst among residents of cities with over 100,000 inhabitants and rural residents (80% of urban residents of 50–100,000 vs. 72% of urban residents of 100–500,000 vs. 76% of rural residents, *p* = 0.008). People with obesity and overweight willingly followed the recommendations of diet modification, as opposed to people with normal body weight (overweight people 77% vs. people with normal body weight 72%, *p* = 0.022).

### 5.3. Limiting Alcohol Consumption

People over 40 reduced their alcohol consumption to a lesser extent after the intervention of a primary care physician than younger people (people over 40 years of age below 89% vs. people 20–39 years of age above 92%, *p* = 0.018). Women and people with arterial hypertension more often followed this recommendation (women vs. men 89% vs. 84%; *p* = 0.003; people with hypertension vs. people without hypertension 87% vs. 83%, *p* = 0.023). Place of residence, lipid profile values, and body weight did not have an impact on the extent of alcohol consumption. Physically working people reduced their alcohol consumption less frequently (manual workers vs. other types of professional activity 80% vs. more than 84%, *p* = 0.0001).

### 5.4. Smoking

People under 50 and over 80 years of age had the greatest difficulty in quitting smoking (<36% and 31%, respectively, *p* = 0.008). Obese and hypertensive people were more likely to quit or reduce smoking (obese people vs. normal body weight 49% vs. 39%, *p* < 0.0001; people with hypertension vs. without 44% vs. 38%, *p* = 0.009). The occupation and the lipid profile values had no effect.

## 6. Discussion

Our study based on 8667 patients and 862 physician-researchers revealed that even 3 months of personalized behavioral interventions resulted in benefits across a variety of important intermediate health outcomes with a significant reduction in metabolic risk factors such as body weight, total cholesterol levels, triglycerides, and LDL cholesterol as well as lowering of peripheral arterial pressure, both systolic and diastolic. The percentage of respondents who fully complied with the doctor’s recommendations and expression with the implemented treatment increased significantly after behavioral interventions. In general, the inhabitants of small towns (50–100,000) and younger persons follow the recommendations the best. The gender and type of work performed did not have statistical significance. The best adherence to the instructions for physical activity was observed in patients with normal body weight and overweight but not obese. Higher values of TCh and LDL cholesterol motivated patients to increase physical activity. People with obesity and overweight willingly followed the recommendations of diet modification, as opposed to people with normal body weight. People under 50 and over 80 years of age had the greatest difficulty in quitting smoking. People over 40 reduce their alcohol consumption to a lesser extent after the intervention of a primary care physician than younger people. Physically working people reduced their alcohol consumption significantly less frequently. In our population, patients over 50 years of age, and especially those over 70, presented poor compliance with dietary recommendations, the worst adherence to lifestyle modification had the patients aged 70–90 years compared to younger patients. People over 40 reduced their alcohol consumption to a lesser extent after the intervention of a primary care physician than younger people. Perhaps one of the reasons might be the fact that the recollection of risk factor information decreased with age, and patients aged ≥ 65 years were fifty percent less likely to recollect risk factor information compared to younger patients [12].

Obese and hypertensive people were more likely to quit or reduce smoking and hypertensive women more often reduced alcohol consumption. Lifestyle intervention programs comprise the first-choice therapy to reduce the cardiovascular risk factors and therefore reduce the risk for heart diseases, one of the main public health problems nowadays [13]. The twelve-week clinical trial of Piovesan et al. included 125 adults who presented at least three of the criteria defined by the revised NCEP ATP III (National Cholesterol Education Program Adult Panel III) for metabolic syndrome (MetS) [13]. The Group Intervention and Individual Intervention patients presented a significant decrease in body mass index, abdominal circumference, and diastolic and systolic arterial pressure after the intervention. The number of diagnostic criteria for MetS decreased significantly. In this study, similarly to ours, the non-pharmacological strategies for changing lifestyle led to a reduction of cardiovascular risk factors [14].

In a randomized controlled trial, Saboya et al. included 72 individuals with MetS aged 30–59 years and randomized them into three groups of multidisciplinary intervention: standard, group, and individual during 12 weeks [5]. The primary outcome was a change in the metabolic parameters and, secondarily, the improvement in quality of life (QOL) measures. Group and individual interventions resulted in a significant reduction in waist circumference, body mass index, as well as systolic blood pressure at 3 months and the improvement of QOL, although it was significantly associated with the physical functioning domain. However, opposite to our study, the authors did not observe statistically significant effects on triglycerides and diastolic blood pressure, including all interventions. In the study of Eriksson et al., 123 subjects from a lifestyle intervention group obtained a significant increase in maximal oxygen uptake, better physical activity, improvement in quality of life, and a significant decrease in body weight, waist and hip circumference, body mass index, waist–hip ratio, systolic and diastolic blood pressure, triglycerides, and glycosylated hemoglobin [15]. Lin et al. performed a systematic review of randomized controlled trials published from January 1985 to June 2014 to evaluate the effectiveness of lifestyle-modification programs on metabolic risks [16]. Among the five trials included, the most commonly applied interventions were diet plans, supervised exercise, health education, individual counseling, behavioral modification, and motivational interviewing. Three-fifths of the studies were nurse-led, and only one of the selected trials was theory-guided. The lifestyle-modification programs effectively reduced triglyceride levels, waist circumference, and systolic blood pressure. However, few trials consistently confirmed the benefits of metabolic risks, and none revealed a significant effect on high-density lipoprotein or fasting blood glucose. The duration of the lifestyle modification programs in the included trials ranged from 4 to 24 weeks, and durations of at least 12 weeks significantly improved quality of life [16]. In order to develop an effective counseling system for the prevention of cardiovascular diseases, the association of a favorably changed lifestyle with improved risk factors was examined. Participants were 7321 office workers aged 30–69 years from in and around Nagoya city. Those who began to eat breakfast and increased their vegetable intake normalized their previously abnormal diastolic blood pressure with more than twice the likelihood (adjusted OR [95% CI] 2.89 [1.29–6.46] and 2.60 [1.18–5.75], respectively). ‘Began to eat breakfast’ was also significantly associated with normalized total cholesterol (TC) (1.84, [1.05–3.21]). ‘Stopped eating till full’ significantly normalized the body mass index (2.03; [1.25–3.28]), uric acid (1.65; [1.07–2.52]) and TC (1.43; [1.04–1.97]). Those who started regular exercise significantly normalized their high–density lipoprotein–cholesterol (HDL-C) abnormality with 1.69 times the likelihood (1.69; [1.24–2.29]), and those who began to walk briskly also improved their TC abnormality (1.85; [1.19–2.89]). HDL-C was normalized with 2.55 times the likelihood in those who quit smoking (2.55; [1.68–3.86]) [17]. Patnode et al. conducted a systematic review to support the U.S. Preventive Services Task Force (USPSTF) in updating its 2012 recommendation on behavioral counseling to promote a healthful diet and physical activity for the primary prevention of CVD in adults without known CVD risk factors [17]. The authors included 88 trials reported in 145 publications. Similarly as in our study there was evidence of statistically significant improvements in systolic blood pressure (mean difference [MD], −1.26 mm Hg [95% confidence interval (CI), −1.77 to −0.75]; k = 22), diastolic blood pressure (MD, −0.49 mm Hg [95% CI, −0.82 to −0.16]; k = 23), low-density lipoprotein cholesterol level (MD, −2.58 mg/dL [95% CI, −4.30 to −0.85]; k = 13), and total cholesterol level (MD, −2.85 mg/dL [95% CI, −4.95 to −0.75]; k = 19) at 6 to 12 months associated with healthful diet and/or physical activity interventions. For adiposity outcomes, interventions were associated with improvements in body mass index (MD, −0.41 kg/m^2^ [95% CI, −0.62 to −0.19]; k = 20), weight (MD, −1.04 kg [95% CI, −1.56 to −0.13]; k = 20), and waist circumference (MD, −1.19 cm [95% CI, −1.79 to −0.59]; k = 17). In accordance with previous reports, our study demonstrated that lifestyle intervention produced beneficial effects on cardiovascular risk factors, especially on weight loss, blood pressure, and lipid profile. A prevention program in primary healthcare with a focus on physical activity, quitting smoking, and diet counseling can favorably influence several risk factors for cardiovascular diseases as well as compliance and satisfaction with treatment. In our study, the implementation of lifestyle counseling also significantly improved compliance and satisfaction with treatment. It is inconclusive whether this improvement might be related to weight loss, blood pressure normalization, quitting smoking, a healthy diet, improvement in the physical condition [18], or both [19].

Maintaining a healthy lifestyle in the secondary prevention of coronary heart disease was thought to be equally important as pharmacotherapy, which serves as an independent factor in reducing cardiovascular morbidity and mortality [20]. Findings have suggested that adhering to a combination of healthy behaviors (non-smoking, moderate alcohol intake, physical activity, and fruit and vegetable consumption) was associated with a lower risk of CVD mortality [21].

## 7. Limitations of the Study

Our review was limited to interventions that were conducted in primary care, or those that we felt may be feasible for primary care. Our study represented an unselected population of patients treated in primary care because of CVD diseases. Lack of randomization is a study limitation. However, using real data gives us the ability to understand how excluded in randomized control trials, individuals react to interventions, which provides a broader insight into the population studied. Another limiting factor concerns the relatively small follow-up period of 12 weeks. Although this is the period normally used in other trials, results of improvement of metabolic parameters might have been maintained if the intervention had lasted longer. The questionnaire and educational intervention did not cover all aspects of lifestyle modification and information about SCORE score, so it is unknown how variables such as resting, sleeping, and mental or social activities could change risk factor profiles.

## 8. Conclusions

As a result of pro-health education in the field of lifestyle modifications, a significant reduction of risk factors for cardiovascular diseases, as well as improved compliance and satisfaction with pharmacological treatment, was observed. Thus, appropriate personalized advice on lifestyle habits should be given to each examinee in a positive, systematic way following the periodic health check-ups in order to reduce the person’s risk and improve the effectiveness of the treatment.

## Figures and Tables

**Table 1:** Characteristics of age and BMI in studied population.

		Mean	SD	95% CI
Age (years)	Women	65.11	11.29	64.76–65.47
	Men	62.69	11.23	62.34–63.04
	Both	63.89	11.32	63.63–64.14
BMI (kg/m^2^)	Women	29.00	4.91	28.85–29.16
	Men	29.32	4.49	29.18–29.46
	Both	29.17	4.70	29.06–29.27

**Table 2:** Comparison of blood pressure between men and women at the first visit.

Blood Pressure The First Visit	Women Mean (±SD)	Men Mean (±SD)	*p*
Systolic	136.91	139.01	<0.0001
(mmHg)	(14.74)	(14.58)	
Diastolic	82.47	84.26	<0.0001
(mmHg)	(9.73)	(9.69)	

*p*-value was calculated by the use of Student *t*-test; *p* < 0.05 was considered as significant.

**Table 3:** Comparison of lipids profile between first and the second visit.

Lipid Fraction	1st Visit Mean (±SD)	2nd Visit Mean (±SD)	*p*
Total cholesterol	209 (±76)	196 (±41)	<0.001
LDL-cholesterol	121(±48)	111(±34)	<0.001
HDL-Cholesterol	55 (±28)	55 (±24)	0.436
Triglicerydes	154 (±81)	140 (±55)	<0.001

*p*-Value calculated by the paired two-sample *t*-tests. The normal distribution of lipid’s profile data was checked by Shapiro–Wilk test.
